# Highly Sensitive and Multiplexed In Situ RNA Profiling with Cleavable Fluorescent Tyramide

**DOI:** 10.3390/cells10061277

**Published:** 2021-05-21

**Authors:** Lu Xiao, Joshua Labaer, Jia Guo

**Affiliations:** Biodesign Institute & School of Molecular Sciences, Arizona State University, Tempe, AZ 85287, USA; lxiao15@asu.edu (L.X.); Joshua.Labaer@asu.edu (J.L.)

**Keywords:** transcriptomics, genomics, fluorescence in situ hybridization, FISH, transcripts

## Abstract

Understanding the composition, regulation, and function of complex biological systems requires tools that quantify multiple transcripts at their native cellular locations. However, the current multiplexed RNA imaging technologies are limited by their relatively low sensitivity or specificity, which hinders their applications in studying highly autofluorescent tissues, such as formalin-fixed paraffin-embedded (FFPE) tissues. To address this issue, here we develop a multiplexed in situ RNA profiling approach with a high sensitivity and specificity. In this approach, transcripts are first hybridized by target-specific oligonucleotide probes in pairs. Only when these two independent probes hybridize to the target in tandem will the subsequent signal amplification by oligonucleotide hybridization occur. Afterwards, horseradish peroxidase (HRP) is applied to further amplify the signal and stain the target with cleavable fluorescent tyramide (CFT). After imaging, the fluorophores are chemically cleaved and the hybridized probes are stripped by DNase and formamide. Through cycles of RNA staining, fluorescence imaging, signal cleavage, and probe stripping, many different RNA species can be profiled at the optical resolution. In applying this approach, we demonstrated that multiplexed in situ RNA analysis can be successfully achieved in both fixed, frozen, and FFPE tissues.

## 1. Introduction

Multiplexed RNA profiling in single cells, in their native spatial contexts, holds great promise to reveal the composition, function, regulation, and interaction of the different cell types in complex biological systems [1]. Microarrays [2] and next-gen sequencing [3,4] allow the analysis of gene expression on a transcriptome scale. However, these approaches require RNA isolation and purification before deciphering their identities and abundances. As a result, the location information of the transcripts is lost. Fluorescence in situ hybridization (FISH) [5] and molecular beacons [6] enable in situ RNA analysis. Nonetheless, these methods can only profile a handful of different transcripts in one sample, as the spectral overlap of commonly available organic fluorophores [7] limits their multiplexing capacity.

To enable multiplexed in situ RNA profiling, combinatorial labeling [8,9,10], reiterative hybridization [11,12,13,14,15], multiplexed error-robust FISH (MERFISH) [16,17,18], sequential hybridization [19,20], and in situ sequencing [21,22] have been developed recently. However, due to their limited sensitivity and specificity, these technologies have not been successfully applied in analyzing formalin-fixed, paraffin-embedded (FFPE) tissues. FFPE tissues are the most common type of archived clinical tissue samples and are routinely examined for disease diagnosis and prognosis [23]. Considering the large number of RNA biomarkers discovered by transcriptome profiling studies, approaches enabling multiplexed in situ RNA analysis in FFPE tissues are urgently needed.

Here, we report a highly sensitive and multiplexed in situ RNA profiling approach. In this approach, RNA targets are stained with horseradish peroxidase (HRP), conjugated oligonucleotides, and cleavable fluorescent tyramide (CFT) with both a high sensitivity and specificity. After imaging, the fluorophores are chemically cleaved, and the oligonucleotide probes are stripped. Through cycles of target staining, fluorescence imaging, fluorophore cleavage, and probe stripping, highly sensitive and multiplexed RNA analysis can be achieved in single cells in situ. We demonstrate that the staining signals can be very efficiently removed by a mild chemical reaction and that the oligonucleotide probes can also be stripped by DNase and formamide solutions with a high efficiency. These two processes maintain RNA integrity. In applying this approach, we show that multiple RNA targets in FFPE and fixed frozen tissues can be unambiguously detected by our approach. With these multiplexed single-cell RNA profiling data, we study the varied cell type compositions and their spatial organizations within tissues. We also demonstrate that this RNA analysis approach can be combined with multiplexed protein imaging technology, which allows multiple RNA and protein molecules to be profiled together in the same tissue in situ.

## 2. Materials and Methods

### 2.1. General Information

Chemicals and solvents were purchased from Sigma-Aldrich (St. Louis, MO, USA) or Ambion (Austin, TX, USA), and were used without further purification, unless otherwise indicated. Bioreagents were purchased from Invitrogen (Carlsbad, CA, USA), unless otherwise noted.

### 2.2. FFPE Tissue Pretreatment

The mouse lung formalin-fixed paraffin-embedded (FFPE) tissue slide was baked for 1 h at 60 °C. Subsequently, the deparaffinization of the slide was performed by 3 times xylene wash, each for 5 min. Then, the slide was immersed 3 times in 100% ethanol at room temperature, each for 1 min. Afterwards, HRP blocking was conducted via a 10 min incubation of the slide in HRP blocking buffer (0.15% H_2_O_2_, 0.1% Triton-X 100 in 1× phosphate buffered saline (PBS)) at room temperature, followed by 3 times 1× PBS wash at room temperature, each for 5 min. After blocking, the slide was incubated with the antigen retrieval buffer (10 M sodium citrate, 0.05% Tween 20, pH 6.0) at 100 °C for 15 min. Finally, the slide was incubated with RNAscope^®^ Protease Plus (Advanced Cell Diagnostics, Newark, CA, USA) for 30 min and washed with 1× PBS 3 times at room temperature, each for 5 min.

### 2.3. Fixed Frozen Tissue Pretreatment

The mouse spinal cord fixed frozen tissue slide was first dehydrated by successive incubations in 50%, 70%, and 100% ethanol at room temperature, each for 5 min. Then, the slide was baked for 10 min at 60 °C. Subsequently, HRP blocking was performed via a 10 min incubation of the slide in HRP blocking buffer at room temperature, followed by 3 times 1× PBS wash at room temperature, each for 5 min. Finally, the slide was incubated with RNAscope Protease IV (Advanced Cell Diagnostics, Newark, CA, USA) for 30 min and washed with 1× PBS 3 times at room temperature, each for 5 min.

### 2.4. Mutliplexed RNA Analysis with CFT

After pretreatment, mouse lung FFPE tissue or mouse spinal cord fixed frozen tissue was first incubated with RNAscope mRNA probe (Advanced Cell Diagnostics, Newark, CA) for 2 h at 40 °C. Then, the slide was treated with RNAscope^®^ Multiplex Fluorescent Reagent Kit v2 (Advanced Cell Diagnostics, Newark, CA, USA), following the manufacturer’s instructions. Signal development was subsequently performed by incubating the slide with 0.25 pmol/uL tyramide-N_3_-Cy5 in amplification buffer (0.0015% H_2_O_2_, 0.1% triton X-100 in 0.1 M boric acid, pH 8.5) at 40 °C for 30 min. Afterwards, the slide was washed 3 times with 1× RNAscope Wash Buffer (Advanced Cell Diagnostics, Newark, CA, USA) at 40 °C, each for 5 min. After imaging, the slide was incubated with cleavage solution (100 mM TCEP in 5× saline sodium citrate (SSC), pH 9.5) at 40 °C for 30 min to remove fluorescence signal. Oligonucleotide probes were then stripped by incubating the slide with 0.5 unit/µL DNase I (Roche Diagnostics, Basel, Switzerland) at room temperature for 1 h. DNase was subsequently quenched by washing the slide with DNase quenching buffer (30% lithium dodecyl sulfate and 30% formamide in Tris-EDTA buffer) at room temperature, each for 10 min. In order to fully strip of the remaining oligonucleotide probes, the slide was then incubated with 70% formamide in 2× SSC at 40 °C for 30 min, followed by 3 times 1× PBS wash at room temperature. The slide was then ready for the next cycle of RNA detection.

### 2.5. Mutliplexed Protein Analysis with CFT

The mouse spinal cord fixed frozen tissue, after pretreatment, was blocked in 1× blocking buffer (1% bovin serum albumin, 0.1% Triton X-100, 10% normal goat serum) at room temperature for 1 h. Then, the slide was incubated with 5 µg/mL of HRP-conjugated primary antibody in 1× blocking buffer at room temperature for 45 min, and subsequently washed 3 times with PBT (0.1% Triton-X 100 in 1× PBS) at room temperature, each for 5 min. Afterwards, signal development was performed by incubating the slide with 10 pmol/uL tyramide-N_3_-Cy5 in an amplification buffer at room temperature for 7 min. After imaging, the slide was incubated with cleavage solution at 40 °C for 30 min to remove fluorescence signal. The slide was then ready for the next cycle of protein detection or mRNA detection. The primary antibodies used include HRP-conjugated rabbit anti-HMGB1 (Thermo Fisher Scientific, Waltham, MA; PA5-22722) and HRP-conjugated mouse anti-hnRNP K (Abcam, Cambridge, United Kingdom; ab204456).

### 2.6. Imaging and Data Analysis

The slide to be imaged was first incubated with GLOX buffer (0.4% glucose and 10 mM Tris HCl in 2× SSC) for 1–2 min at room temperature. Then, the slide was imaged in GLOX solution (0.37 mg/mL glucose oxidase and 1% catalase in GLOX buffer).

Both the FFPE slides and the fixed frozen slides were imaged under a Nikon Ti-E epifluorescence microscope equipped with a 20× objective. Images were taken using a CoolSNAP HQ2 camera (Tokyo, Japan). DAPI images were taken using the C-FL DAPI HC HISN filter. Cy5 images were taken using the Chroma 49009 filter. Imaging, processing, and pseudo-color assignment were processed by ImageJ [24]. Cell segmentation and spot counting were processed by Cell Profiler [25]. The spots detected in varied cycles with a distance less than 320 nm (2 pixels) were considered as reappearing spots. tSNE maps and Phenograph clustering were generated from CYT (https://www.c2b2.columbia.edu/danapeerlab/html/cyt.html, accessed on 5 January 2020) [26].

## 3. Results

### 3.1. Platform Design

**Figure 1:**
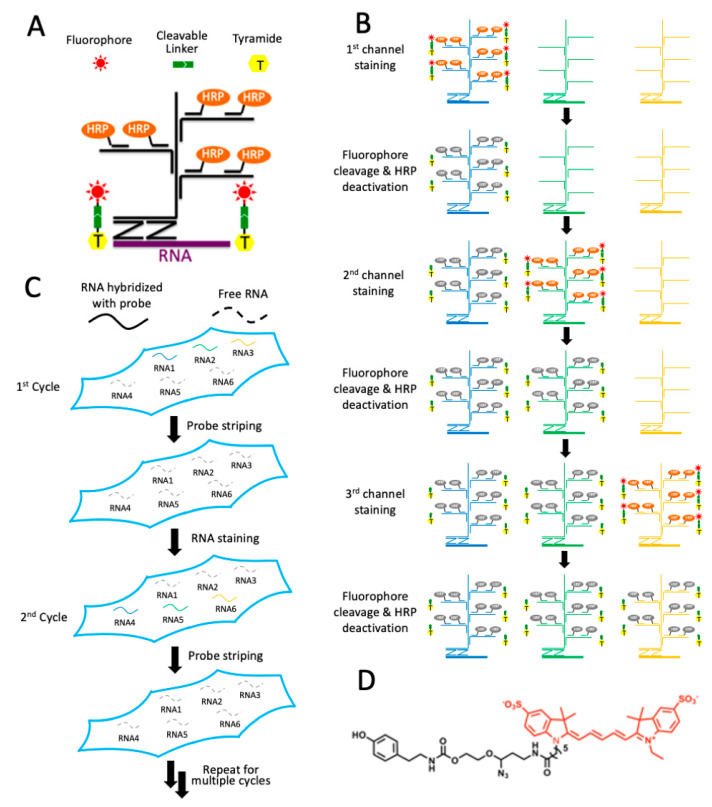
Highly sensitive and multiplexed in situ RNA profiling with cleavable fluorescent tyramide (CFT). (**A**) In each cycle, multiple transcripts of interest are hybridized by oligonucleotide probes in pairs, which recruit oligonucleotide amplification probes. With further signal amplification by horseradish peroxidase (HRP), the target RNA is stained with CFT. (**B**) After imaging, the fluorophores are chemically cleaved and HRP is deactivated, allowing other RNA targets to be stained in the same cycle. (**C**) After all the RNA targets are stained in a cycle, all the hybridized probes are stripped. Through reiterative cycles of RNA staining and probe stripping, a large number of transcripts can be profiled in single cells in situ. (**D**) Structure of CFT, tyramide-N_3_-Cy5.

As shown in Figure 1, each staining cycle of our multiplexed RNA imaging technology is composed of six major steps. First, the RNA targets are hybridized by ~20 pairs of oligonucleotide probes (Figure 1A). To ensure staining specificity, only when both probes in each probe pair hybridize to the transcript in close proximity would the subsequent signal amplification by oligonucleotide hybridization be initiated. These target hybridization and signal amplification processes are carried out simultaneously for all the different RNA targets in each cycle. Second, the first RNA target is stained with horseradish peroxidase (HRP), conjugated oligonucleotides, and CFT. Third, the specimen is imaged to generate quantitative single-cell in situ RNA expression profiles. Fourth, without damaging the RNA integrity, the fluorophores are efficiently removed using a mild chemical reaction and HRP is deactivated by H_2_O_2_. Fifth, step two to four may be repeated to stain other RNA targets in the first cycle (Figure 1B). Finally, all the hybridized probes are stripped by DNase and formamide solution so that the same signal amplification oligonucleotide probes can be applied in the next cycle. Through reiterative cycles of target staining, fluorescence imaging, fluorophore removal, HRP deactivation, and probe stripping, many different RNAs can be visualized in single cells within the same specimen (Figure 1C). With varied RNA species, stained in different cycles, a total of M × N RNA targets can be profiled in one specimen, where M is the number of targets detected in each cycle and N is the number of cycles. With the same set of RNA species visualized in different cycles, each RNA can be identified as a unique fluorescence sequence barcode. In this case, a total of M^N^ RNA species can be profiled in situ, where M is the number of staining in each cycle and N is the cycle number.

### 3.2. Efficient Fluorphore Cleavage

**Figure 2:**
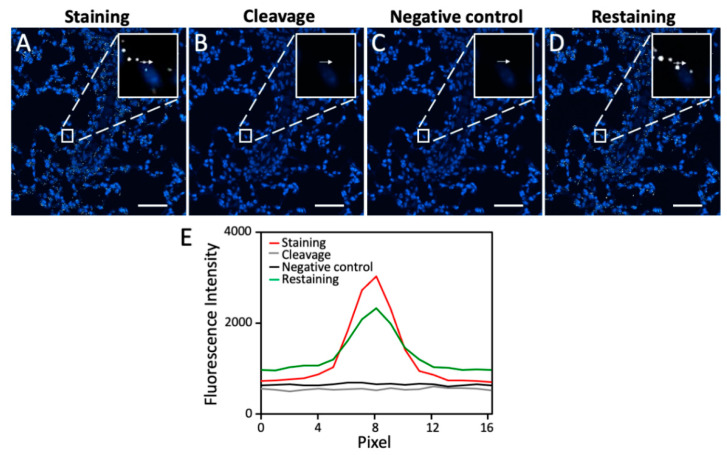
(**A**) mRNA PPIB in FFPE mouse lung tissue is hybridized with the target recognition and signal amplification probes and then stained with tyramide-N_3_-Cy5. (**B**) The fluorophore is cleaved by TCEP at 40 °C for 30 min. (**C**) After probe stripping with 1 h DNase treatment at room temperature and 30 min 70% formamide treatment at 40 °C, the tissue is incubated with the signal amplification probes and tyramide-N_3_-Cy5, in the absence of the target recognition probes. (**D**) After 12 h of TCEP treatment at 40 °C, 24 h of DNase treatment at room temperature, and 12 h of 70% formamide treatment at 40 °C, mRNA PPIB in the same FFPE mouse lung tissue is rehybridized with the target recognition and signal amplification probes and then stained with tyramide-N_3_-Cy5. (**E**) Fluorescence signal intensity corresponding to the arrow positions in (**A**–**D**). Scale bar, 50 µm.

One critical requirement for the success of this multiplexed RNA imaging approach is to efficiently cleave the fluorophores so that the signals generated from the previous cycles will not result in false positive results in the following cycles. To assess the fluorophore cleavage efficiency, we stained mRNA PPIB in an FFPE mouse lung tissue using tyramide-N_3_-Cy5 (Figure 1D). This CFT molecule has been recently developed in our laboratory for multiplexed protein imaging [27]. We have demonstrated that tyramide-N_3_-Cy5 can be successfully converted by HRP to a short-lived reactive radical. This covalently binds to the tyrosine residues on the proteins proximal to the target. Since HRP is also required in this sensitive RNA imaging approach, we adopted tyramide-N_3_-Cy5 as the staining reagent. The RNAscope oligonucleotide probes enable the RNA detection in FFPE tissues with both high sensitivity and specificity [28]. Thus, in this study we applied RNAscope probes to recognize the RNA target and amplify the signals by oligonucleotide hybridization. After staining, mRNA PPIB was unambiguously detected in the FFPE lung tissue (Figure 2A). Following the incubation of the stained tissue with TCEP at 40 °C for 30 min, almost all of the fluorescence signals were removed (Figure 2B,E). These results suggest that the RNA FISH signals generated by CFT can be efficiently erased by a fluorophore cleavage reaction within a short time.

### 3.3. Efficient Probe Stripping

Another crucial requirement for this multiplexed RNA imaging method to succeed is to efficiently strip the hybridized probes at the end of each analysis cycle. In this way, when the same set of signal amplification probes is applied in the later cycles, the RNA targets profiled in the previous cycles will not be restained. To evaluate the probe stripping efficiency, we first applied DNase at room temperature for one hour to degrade the oligonucleotide probes, followed by probe dehybridization with 70% formamide at 40 °C for 30 min. In the absence of any target recognition probes, the signal amplification oligonucleotide probes and CFT were applied again on the same mouse lung tissue. However, no further signal increase was observed (Figure 2C,E). These results indicate that DNase and the formamide solution can efficiently strip the hybridized probes.

### 3.4. RNA Integrity Maintained during Fluorophore Cleavage and Probe Stripping

This multiplexed RNA imaging approach also requires that the fluorophore cleavage reaction and the probe stripping process maintain the RNA integrity so that RNA targets can be successfully detected in subsequent cycles. To evaluate whether the RNA integrity is maintained during these two processes, we incubated the stained tissue with TCEP at 40 °C for 12 h, DNase at room temperature for 24 h, and 70% formamide at 40 °C for 12 h. Then, PPIB mRNA was stained again in the same tissue. The obtained staining patterns and signal intensities are similar to the ones generated in the first cycle (Figure 2D,E). These results suggest that the RNA integrity is maintained during the fluorophore cleavage reaction and the probe stripping process.

### 3.5. Multiplexed In Situ RNA Profiling in FFPE Tissues

**Figure 3:**
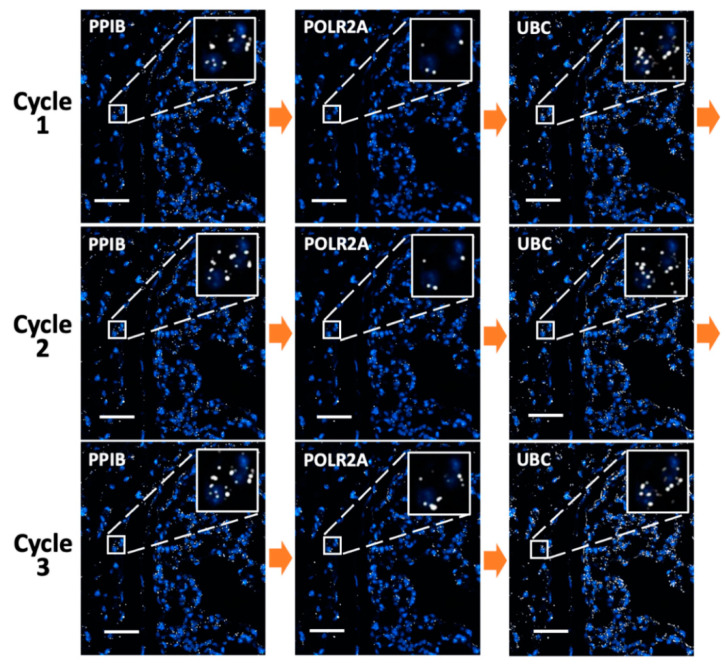
mRNA PPIB, POLR2A, and UBC in an FFPE mouse lung tissue are sequentially stained with tyramide-N_3_-Cy5 in three reiterative cycles. Scale bar, 50 μm.

To demonstrate the feasibility of applying this approach in multiplexed in situ RNA analysis in FFPE tissues, we stained mRNA PPIB, POLR2A, and UBC reiteratively using tyramide-N_3_-Cy5 in the same mouse lung tissue (Figure 3). In each cycle, the three mRNA molecules were stained sequentially and each target was stained three times in three reiterative cycles. For all the RNA targets, the staining patterns and copy numbers obtained in the three analysis cycles were similar. Almost no signal leftover generated in the previous cycles was observed as false-positive signals in the following cycles. As a result, every mRNA molecule can be specifically stained and accurately quantified, regardless of the expression levels of the RNA profiled before. These results suggest that our approach can be successfully applied to multiplexed in situ RNA analysis in FFPE tissues.

**Figure 4:**
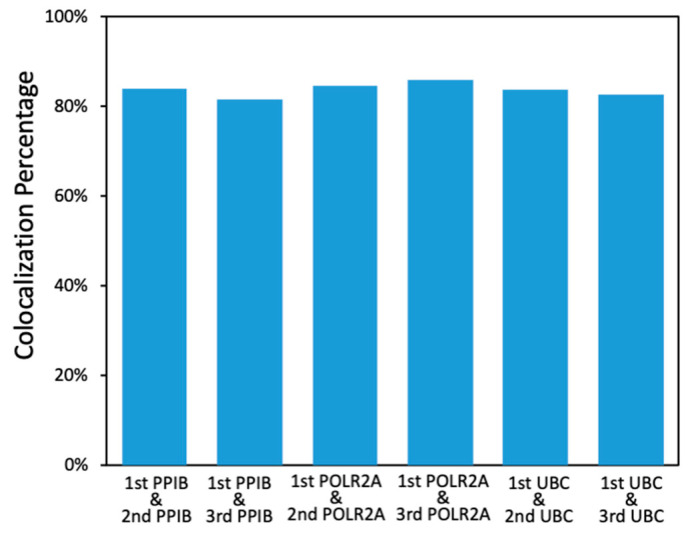
Percentage of the PPIB, POLR2A, and UBC spots identified in the first cycle that reappeared in the following cycles.

As all hybridized probes are efficiently stripped at the end of each cycle, our approach allows the same RNA molecule to be stained multiple times in reiterative analysis cycles. With the RNA targets chemically crosslinked to other biomolecules in the specimen, their cellular locations will remain in place throughout different analysis cycles. Through reiterative staining, the identity of each RNA molecule can be deciphered by a unique fluorescence sequence barcode. In this way, the total number of RNA species that can be quantified in one specimen will increase exponentially with the cycle number. It has been demonstrated that with around 78% of spots identified in the previous cycle that reappear in the next cycle, RNA molecules can be successfully identified with a fluorescence sequence barcode [20]. In our approach, for every RNA target in all the cycles, over 80% of the spots detected in the first cycle were also identified in the following cycles (Figure 4). These results suggest that our approach has the potential to enable spatial transcriptomics analysis in FFPE tissues by assigning each RNA species a unique fluorescence sequence barcode.

### 3.6. Multiplexed In Situ RNA Profiling in Fixed Frozen Tissues

**Figure 5:**
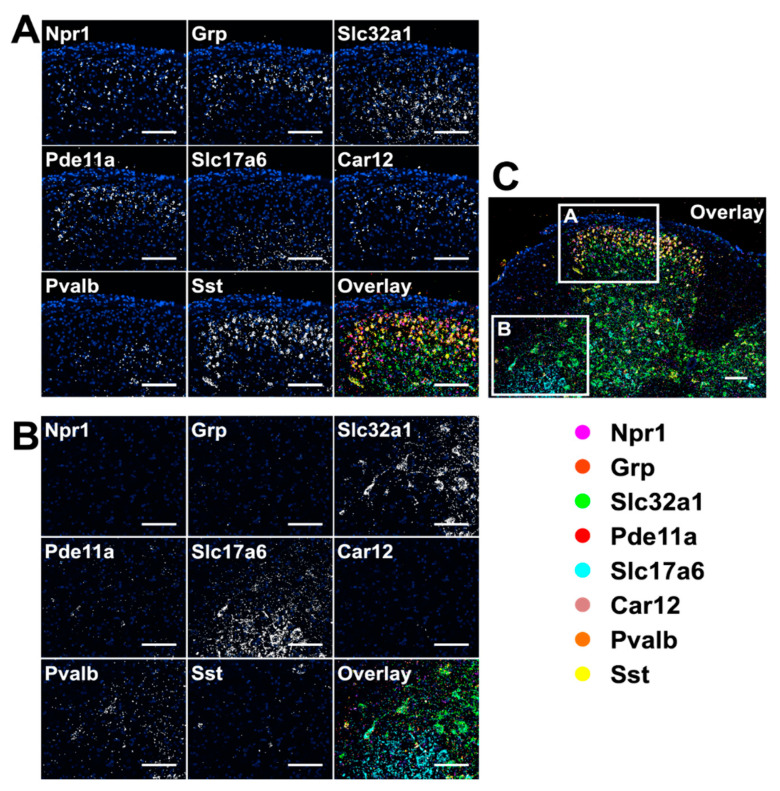
Eight different mRNA in a fixed frozen mouse spinal cord tissue are stained sequentially with tyramide-N_3_-Cy5. (**A**,**B**) are the different regions of the overlay image (**C**). Scale bar, 100 µm.

**Figure 6:**
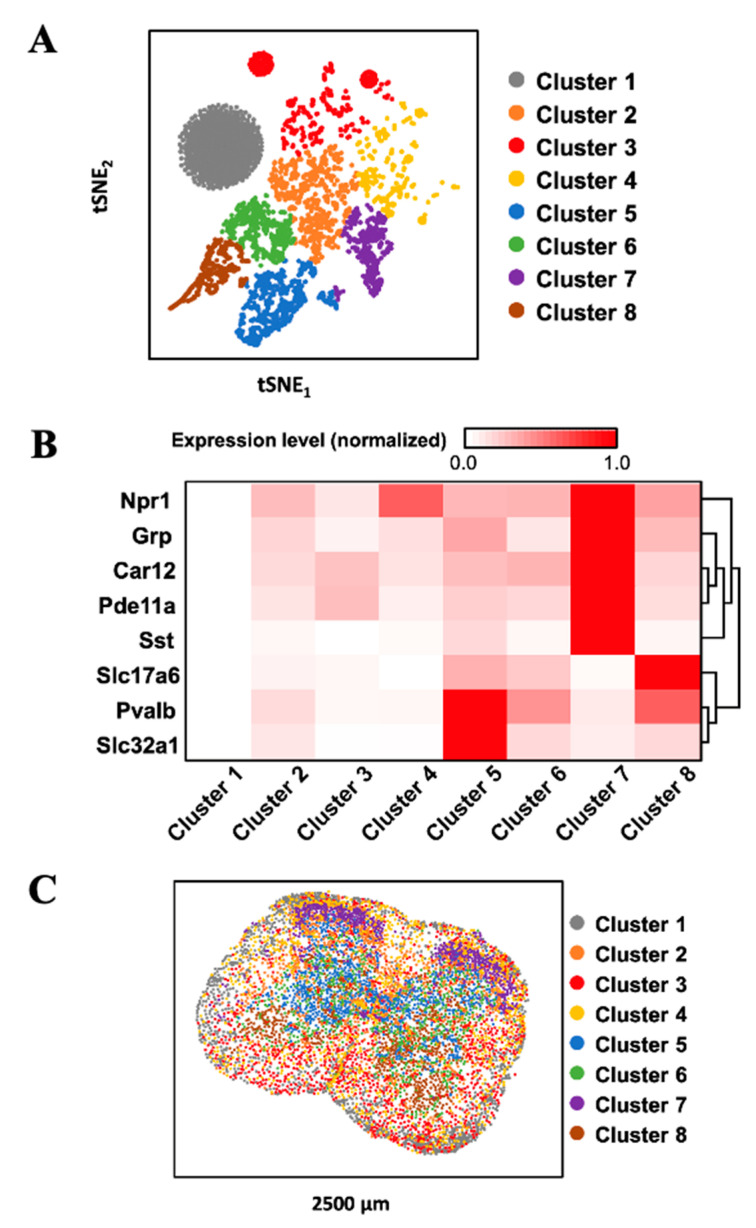
(**A**) Based on their mRNA expression patterns, the cells in a fixed frozen mouse spinal cord tissue are partitioned into 8 clusters. (**B**) The mRNA expression patterns in the different cell clusters. (**C**) Anatomical locations of the individual cells from the distinct clusters in the spinal cord tissue.

To demonstrate the feasibility of applying our approach for multiplexed RNA analysis in fixed frozen tissues, we stained eight RNA species using tyramide-N_3_-Cy5 in mouse spinal cord tissues (Figure 5). Through reiterative cycles of target staining, fluorescence imaging, fluorophore removal, HRP deactivation, and probe stripping, mRNA Npr1, Grp, Slc32a1, Pde11a, Slc17a6, Car12, Pvalb, and Sst were unambiguously detected in the same tissue. With these multiplexed single-cell in situ RNA profiling results, we explored the cell heterogeneity and their spatial organization in the mouse spinal cord (Figure 6). In the examined spinal cord tissue, we calculated the RNA copy numbers in more than 5000 single-cells. We then partitioned those individual cells into eight cell clusters (Figure 6A) using the software viSNE [26] based on their single-cell RNA expression profiles (Figure 6B). By mapping the eight cell clusters back to their native positions in the tissue (Figure 6C), we observed that the varied subregions of spinal cord are composed of cells from different clusters. For instance, the lateral and ventral funiculus are dominated by cell clusters one, two, and four. Within the dorsal and ventral horns, cell clusters two and seven appear on the edges, while cell clusters five, six, and eight are the major cell types in the inner areas. These results suggest that our approach can be successfully applied to multiplexed in situ RNA profiling in fixed frozen tissues, and that it also allows the exploration of varied cell type compositions and their spatial organizations in the tissues.

### 3.7. Combined RNA and Proteins In Situ Profiling

**Figure 7:**
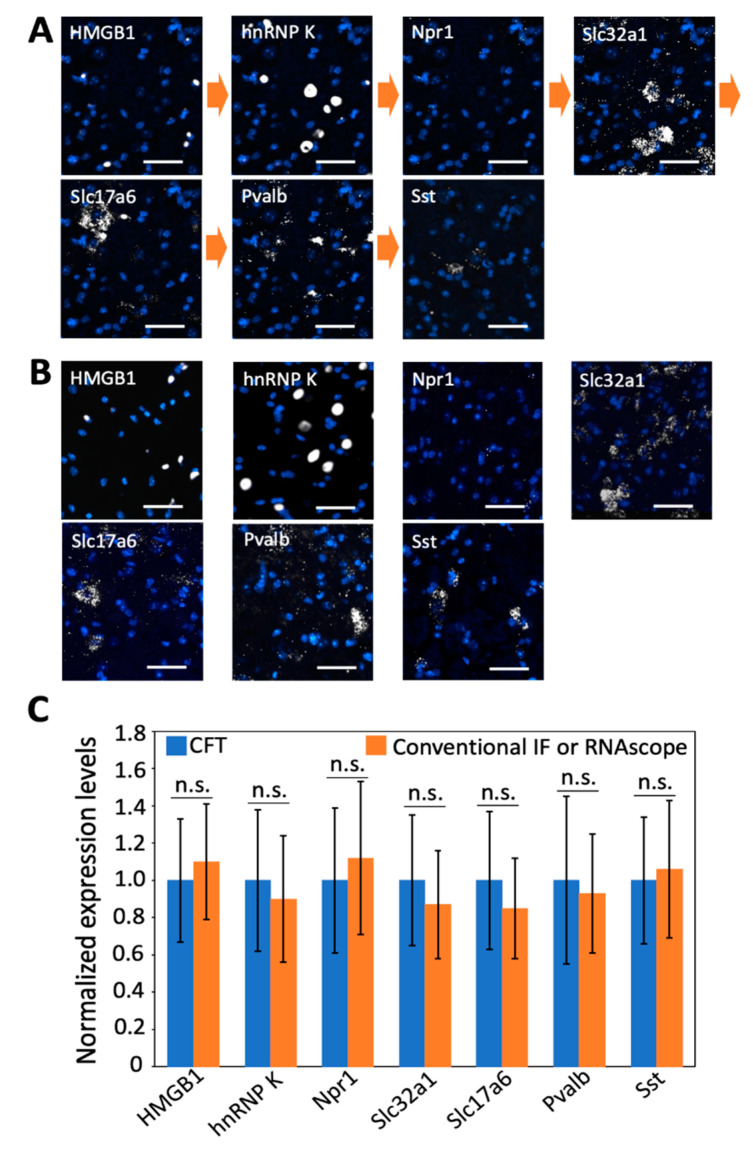
(**A**) Proteins HMGB1 and hnRNP K together with mRNA Npr 1, Slc32a1, Slc17a6, Pvalb, and Sst are stained sequentially with tyramide-N_3_-Cy5 in a fixed frozen mouse spinal cord tissue. (**B**) The same two proteins and five mRNA are stained in seven different fixed frozen mouse spinal cord tissues by conventional immunofluorescence (IF) or RNAscope. (**C**) Comparison of the normalized single-cell expression levels obtained in (**A**,**B**) (*n* = 30 cells). Error bars generated using standard error of the mean. n. s., *p* > 0.4. Scale bar, 40 µm.

Integrated in situ analysis of RNA and proteins in the same specimen is of increasing importance in studies of gene expression regulation [29] and disease diagnosis [30]. Our laboratory recently developed cleavable fluorescent probes [27,31,32,33] for multiplexed protein imaging and demonstrated that these probes enable a large amount of different proteins to be accurately quantified in their native cellular contexts in single cells. To evaluate whether the multiplexed RNA and proteins in situ analysis can be combined in the same tissue using CFT, we stained two proteins and five mRNA sequentially using tyramide-N_3_-Cy5 in a mouse spinal cord tissue (Figure 7A). This tissue was first incubated with HRP-conjugated antibodies and tyramide-N_3_-Cy5 to stain the protein HMGB1. After fluorophore cleavage and HRP deactivation, the protein hnRNP K was stained using the same approach. Subsequently, HRP-conjugated oligonucleotides and tyramide-N_3_-Cy5 were applied to detect mRNA Npr1, Slc32a1, Slc17a6, Pvalb, and Sst in reiterative analysis cycles. We also performed the control experiments by staining the same seven targets in different tissues using conventional immunofluorescence and RNAscope approaches (Figure 7B). The staining patterns and expression levels obtained by our method and conventional approaches closely resemble each other (Figure 7C). These results suggest our approach allows the direct visualization and quantitative analysis of multiple RNA and proteins together in the same specimen.

## 4. Discussion

In summary, we developed cleavable fluorescent tyramide for highly sensitive and multiplexed in situ RNA profiling. This novel approach enables RNA visualization in FFPE and fixed frozen tissues and also allows the combined analysis of RNA and proteins in the same specimen. Compared with the existing multiplexed RNA imaging technologies, our approach dramatically improves the detection sensitivity by using signal amplifications with both the oligonucleotide hybridization and HRP. These amplified signals enable transcripts to be unambiguously detected and accurately profiled with a much shorter exposure time and an objective with low magnification. As a result, our approach significantly reduces the assay time and enhances the sample throughput. More importantly, our approach also has a high staining specificity by hybridizing the target with oligonucleotides in pairs. Due to its high specificity and sensitivity, our approach is currently the only method that has been successfully applied for multiplexed in situ RNA profiling in FFPE tissues.

The two factors that determine the multiplexing capacity of this approach include the number of analysis cycles and the number of RNA profiled in each cycle. We have demonstrated that the RNA integrity is maintained after the treatment with TCEP for 12 h, DNase for 24 h, and 70% formamide for 12 h. These results suggest that at least 12 cycles can be carried out on the same specimen. In each cycle, four or five different RNA targets can be stained with varied fluorophores and imaged simultaneously. Therefore, we anticipate that this technology could profile over 50 distinct RNA species in the same specimen if different RNA targets are stained in different cycles. When the same set of RNA species is stained throughout all the cycles, this approach has the potential to quantify thousands of varied RNA targets within eight cycles (4^8^ = 65,536) in the same tissue.

In addition to RNA and protein analysis, our reiterative staining approach can also be applied to highly sensitive in situ DNA [34] and metabolic analysis [35]. By integrating these applications, the combined spatial genomics, transcriptomics, proteomics, and metabolomics analysis can be achieved in the same tissue at the optical resolution. This ultrasensitive and comprehensive molecular imaging platform will bring new insights into systems biology and precision medicine.

## Data Availability

All the data are included in the manuscript.
